# Drug susceptibility profiling and genetic determinants of drug resistance in *Mycobacterium simiae* isolates obtained from regional tuberculosis reference laboratories of Iran

**DOI:** 10.1371/journal.pone.0267320

**Published:** 2022-08-12

**Authors:** Sara Daneshfar, Azar Dokht Khosravi, Mohammad Hashemzadeh

**Affiliations:** 1 Infectious and Tropical Diseases Research Center, Health Research Institute, Ahvaz Jundishapur University of Medical Sciences, Ahvaz, Iran; 2 Department of Microbiology, Faculty of Medicine, Ahvaz Jundishapur University of Medical Sciences, Ahvaz, Iran; 3 Iranian Study Group on Microbial Drug Resistance, Tehran, Iran; The Foundation for Medical Research, INDIA

## Abstract

**Background:**

Among Non-tuberculous mycobacteria (NTM) which generally cause opportunistic infections, especially in immunocompromised hosts, *Mycobacterium simiae* (*M*. *simiae*) is one of the most important NTM, associated with pulmonary disease. The main concern about *M*. *simiae* infections is the extreme resistance of this NTM to antibiotics. There are limited studies about drug susceptibility testing (DST) and the causes of drug resistance in *M*. *simiae*. Hence, the current study aimed to identify the *M*. *simiae* isolates and to assess the drug resistance of the isolates using phenotypic and molecular methods.

**Materials and methods:**

In this study, 50 clinical pulmonary isolates suspected of NTM were collected from regional tuberculosis reference laboratories in Iran. The isolates were identified as *M*. *simiae* by using standard biochemical tests and molecular methods. DST was performed for identified *M*. *simiae* isolates and additional 35 *M*. *simiae* isolates from the department archive, against eight drugs. The mutations in *gyrA*, *gyrB*, and *rrl* genes in clarithromycin and moxifloxacin resistant isolates were investigated by polymerase chain reaction (PCR) followed by sequencing.

**Results:**

Out of 50 suspected NTM isolates, 25 isolates were detected as *M*. *simiae* species based on the biochemical tests, and 18 isolates were verified based on the *rpoB* gene sequence analysis to achieve a total of 53 isolates when the archive isolates were included. DST results showed that all 53 isolates were resistant to isoniazid, rifampin, and clofazimine. The rate of resistance to ethambutol and linezolid were 34 (64%), and 40 (76%) respectively. The highest susceptibility rate was demonstrated for amikacin 53 (100%) and clarithromycin 45(85%), followed by moxifloxacin 35(66%). Sequence analysis showed mutations in positions 2058 and 2059 of the *rrl* gene, as well non-synonymous mutation at codons 389, 444, and 571 of the *gyrB* gene. Sequence analysis showed no mutation in the *gyrA* gene. drug-resistant isolates with mutations showed higher MICs compared to non-mutant resistant isolates.

**Conclusions:**

This study revealed amikacin, clarithromycin, and moxifloxacin as the most effective antibiotics. However, since *M*. *simiae* exhibited a high level of antibiotic resistance *in vitro*, therefore, species identification and determining the antibiotic susceptibility pattern of the isolates are essential before treatment.

## Introduction

Non-tuberculous mycobacteria (NTM) are a group of bacteria that belongs to the genus of mycobacteria. NTM are generally referred to as environmental bacteria with wide distribution in natural resources however, a number of them are opportunistic pathogens and can cause serious disease, especially in immunocompromised individuals [1]. NTM infections are one of the important causes of death due to tuberculosis (TB) treatment failure [2]. Non-tuberculous mycobacteria which include about 200 known species are classified into two major groups, including slow-growing mycobacteria (SGM), and fast-growing mycobacteria (RGM), based on growth rate [1–3]. One of the most frequent NTM is *Mycobacterium simiae* (*M*. *simiae*) which has been recognized as an opportunistic pathogen and health concern in various areas throughout the world, including Iran [3, 4]. *M*. *simiae* is a photochromogenic, slow-growing NTM that was first isolated from Cercopthecus aethiops and Macacus rhesus monkeys in 1965 [5–7]. Although *M*. *simiae* is initially isolated from natural environments such as water and soil, it can cause a wide range of asymptomatic diseases to diffuse and fatal infections in humans. It has the potential to induce infections in a variety of body organs and is regarded as the most significant pathogens in NTM among patients with underlying diseases such as diabetes mellitus, cystic fibrosis, and particularly pulmonary disease [3, 8]. In a recent meta-analysis study, Nasiri et al. [3], reported a 25% prevalence of this bacterium in Iran. The incidence has even been reported as up to 40% by Lotfi et al in the latest publication [5]. The similarity between the biochemical criteria of *M*. *simiae* and *M*. *tuberculosis* including having a positive niacin test might lead to laboratory misinterpretation [9]. Identification of isolated NTM from clinical specimens to the species level can help to control infections caused by these opportunistic pathogens in medical centers and the community [10, 11]. Today, precise detection of these bacteria to the species level with the application of phenotypic tests is difficult, time-consuming, and inaccurate. Therefore, many researchers use more sophisticated molecular methods such as sequencing to detect NTM [11]. *M*. *simiae* exhibits intrinsic or acquired resistance to various antibiotics, as well as a notable level of resistance to first-line anti-TB drugs. Treatment of this bacterium has become a serious challenge for many physicians due to various resistance mechanisms, some of which are still unknown [5, 9, 12]. Cotrimoxazole, moxifloxacin, and clarithromycin are considered to be the most efficient antibiotics against this pathogen [9], although, in previous studies, the resistance of *M*. *simiae* has been reported to fluoroquinolones (FQs) and macrolides [5, 13]. There is insufficient information to evaluate the association between *in vitro* susceptibility and *in vivo* treatment outcomes for the majority of the drugs [5, 9]. Drug resistance among NTMs is often due to increased drug efflux, decreased drug uptake, increased drug metabolism, or decreased drug sequestration as well as mutations in the genome [14]. However, data on the frequency of genetic mutations associated with FQs and macrolide resistance determination regions in *M*. *simiae* clinical isolates are still limited. This study aimed to identify *M*. *simiae* strains from NTM isolated from patients referred to certain Regional Tuberculosis Reference Laboratories in Iran, by phenotypic and molecular methods, and to determine the resistance profile of the isolates against 8 antibiotics by microbroth dilution method. Moreover, the mutations in genes involved in resistance to macrolides and FQs were also investigated.

## Materials and methods

### Ethics statement

This research was approved by the Ethics Committee of the Ahvaz Jundishapur University of Medical Sciences, Ahvaz, Iran (No: IR.AJUMS.MEDICINE.REC.1399.018) based on Deceleration of Helsinki 2013. Written informed consent was obtained from all patients.

### Sample collection and phenotypic identification

During one year, from February 2019 to February 2020, 50 clinical isolates of pulmonary origin (10 Broncho-alveolar lavage [BAL] and 40 sputum) which belonged to 29 male (58%), and 21 female (42%) patients, suspected of NTM were collected from patients referred to the selected Regional TB Reference laboratories of Iran, including Khuzestan (15 isolates/30%), Kermanshah (14 isolates /28%), Tehran (13 isolates /26%) and Fars (8 isolates /16%). Table 1, describes the patients’ archive information. Additionally, 35 *M*. *simiae* strains from the department archive were also included. Lowenstein-Jensen (LJ) media (Merck/Germany) was used to cultivate all of the strains. Phenotypic characteristics such as pigment production, colony characteristics, acid-fast staining, and biochemical tests including niacin production, semi-quantitative catalase test, tween 80 hydrolysis, arylsulfatase test, urease, stable heat catalase (pH 7, 68°C), and nitrate reduction test were accomplished [15].

**Table 1:** Clinical details of the patients with NTM-Positive culture.

Line	Isolates	Previous medical history	Sex	Sample source	Clinical presentations
**1**	NTM1	HIV	Male	Sputum	Productive cough
**2**	NTM2	Normal	Female	BAL	Productive cough, Fever
**3**	NTM3	HIV	Male	Sputum	Productive cough, Body weight loss
**4**	NTM4	COPD	Male	Sputum	Fever, Body weight loss
**5**	NTM5	Normal	Male	Sputum	Fever, cough
**6**	NTM6	COPD	Male	Sputum	Fever, cough
**7**	NTM10	Normal	Female	Sputum	Productive cough, Fever
**8**	NTM11	Treated tuberculosis	Male	Sputum	Fever
**9**	NTM12	HIV	Male	Sputum	Productive cough, Body weight loss, thoracic pain
**10**	NTM13	Stomach cancer	Female	Sputum	Fever
**11**	NTM14	Normal	Male	Sputum	Fever, cough
**12**	NTM16	Treated tuberculosis	Female	Sputum	Productive cough
**13**	NTM17	Normal	Female	Sputum	Productive cough, Fever
**14**	NTM20	COPD	Male	Sputum	Fever, Cough
**15**	NTM23	HIV	Female	Sputum	Productive cough
**16**	NTM24	Normal	Male	Sputum	Productive cough, Body weight loss
**17**	NTM25	Treated tuberculosis	Male	Sputum	Fever, Cough
**18**	NTM26	Normal	Male	Sputum	Productive cough, Fever
**19**	NTM27	Respiratory failure	Female	Sputum	Productive cough
**20**	NTM28	HIV	Male	BAL	cough
**21**	NTM30	Normal	Female	Sputum	Fever, Body weight loss
**22**	NTM32	Breast cancer	Female	Sputum	Fever, Cough
**23**	NTM34	Normal	Female	Sputum	Productive cough
**24**	NTM35	COPD	Male	Sputum	Local pain, Fever
**25**	NTM36	Diabetic	Female	Sputum	Fever, Cough
**26**	NTM38	HIV	Male	Sputum	Fever
**27**	NTM40	Treated tuberculosis	Female	Sputum	Fever, Cough
**28**	NTM41	Normal	Female	Sputum	Productive cough, Fever
**29**	NTM43	COPD	Male	Sputum	Fever, Thoracic pain
**30**	NTM49	Diabetic	Male	BAL	Fever, Cough
**31**	NTM53	Diabetic	Male	Sputum	Fever, Cough
**32**	NTM54	Normal	Female	Sputum	Cough
**33**	NTM57	Stomach cancer	Female	Sputum	Fever, Cough
**34**	NTM58	HIV	Male	Sputum	Cough
**35**	NTM59	COPD	Female	Sputum	Fever, Cough
**36**	NTM63	HAV	Male	Sputum	Cough
**37**	NTM64	Normal	Female	BAL	Productive cough, Fever
**38**	NTM85	Normal	Female	Sputum	Productive cough, Fever
**39**	NTM86	Dialysis	Male	Sputum	Cough
**40**	NTM87	COPD	Male	BAL	Body weight loss
**41**	NTM96	Immunocompromised	Female	BAL	Productive cough
**42**	NTM99	COPD	Female	Sputum	Fever, cough
**43**	NTM100	Open heart surgery	Male	Sputum	Productive cough
**44**	NTM102	Normal	Male	BAL	Productive cough
**45**	NTM103	COPD	Male	Sputum	Productive cough, fever
**46**	NTM104	Treated tuberculosis	Male	Sputum	Fever, Cough
**47**	NTM110	Normal	Female	BAL	Productive cough
**48**	NTM116	Ranal failure	Male	BAL	Productive cough
**49**	NTM136	HCV, HIV	Male	Sputum	Productive cough
**50**	NTM137	Normal	Male	BAL	Fever, Cough

### Molecular identification

#### DNA extraction

Based on phenotypic and biochemical criteria, 25 mycobacterial clinical isolates were identified as *M*. *simiae*. DNA extraction from 25 isolates was performed by the boiling method as previously stated [16]. The concentration of the extracted DNA was measured at 260 nm using a Nanodrop (Thermo Fisher Scientific, Waltham, MA, USA).

#### Species identification

A 750-bp fragment of the *rpoB* gene was amplified using primers MycoF (5′—GGCAAGGTCACCCCGAAGGG-3′) and MycoR (5′ -AGCGGCTGCTGGGTGATCATC- 3′), as described by Adékambi et al). Table 2 ([17]. A 50-μL reaction mixture comprising 10× PCR buffer (5 μL), deoxynucleotide triphosphate (dNTP; 0.2 mM), MgCl2 (1.5 mM), each primer (0.2 μM), Taq polymerase (2.5 Unit), and 10 ng template DNA (5 μL) was prepared. The cycling program was adjusted as follows: initial denaturation at 95°C for 5 min, followed by 35 cycles of denaturation at 95°C for 45 seconds, annealing at 62°C for 45 seconds, and extension at 72°C for 40 seconds, with a final extension at 72°C for 5 min. The expected amplicons were separated using electrophoresis (70 V, 45 min) on 1.5% agarose gel (EMD Millipore, Billerica, MA, USA) and stained with the SYBR® Safe DNA Gel Stain (Thermo Fisher Scientific). The DNA bands were observed by a gel documentation system (Uvidoc, Jencons Scientific Inc, Cambridge, UK) [18]. The PCR products were sequenced using the ABI PRISM 7500 Sequence Detection System (Applied Biosystems, Foster City, CA, United States). BLAST was used to verify the *rpoB* gene sequences for each isolate. The sequences of the *rpoB* gene for each isolate were aligned separately and compared with all existing relevant sequences of mycobacteria recovered from the GenBank database using the MEGAX program. Percentages of similarity between sequences of each gene were determined by comparing sequences to an in-house database of *rpoB* sequences. Phylogenetic trees were obtained from DNA sequences using the Neighbor-Joining (NJ) method and Kimura’s two-parameter (K2P) distance correction model with 1000 bootstrap replications supported by the MEGAX software (http://www.megasoftware.net) [19].

**Table 2:** Oligonucleotide primers used in PCR and sequencing.

Gene	Primer	Sequence	PCR amplicon size	Reference
*rpoB*	*mycoF*	5’-GGCAAGGTCACCCCGAAGGG-3’	762	13
	*mycoR*	5’-AGCGGCTGCTGGGTGATCATC-3’		
*gyrA*	*gyrA F*	5’-AYTCYGYCGAMCGGATCGAG-3’	459	This study
	*gyrA R*	5’-GCACCCGGCCGTCATAGTTG-3’		
*gyrB*	*gyrB F*	5’-TGGGCAACGCATCGGTGCGA-3’	762	18
	*gyrB R*	5’-AGGGATCCATGGTGGTCTCC-3’		
*rrl*	*rrl F*	5’-CGGGAWYCGGYCGCAGAAC-3’	1110	This study
	*rrl R*	5’-CCAGGTCTGGCCTATCRAWC-3’		

### Drug susceptibility testing (DST)

The minimum inhibitory concentration (MIC) of the antibiotics for the *M*. *simiae* isolates was determined by using the broth microdilution method and interpreted according to the Standard Clinical and Laboratory Standards Institute (CLSI) recommendations [20]. Powdered forms of rifampin, isoniazid, ethambutol, linezolid, clofazimine, clarithromycin, moxifloxacin, and amikacin antibiotics were purchased from Sigma-Aldrich Company and were freshly prepared based on the manufacturer’s guidelines. To achieve the required dilution, a suitable amount of antibiotic stock was added to Middle Brook 7H9 broth (Fluka, Switzerland) containing 2 ml of glycerol and 100 mL of oleic acid/dextrose/catalase (OADC) growth supplement (Sigma-Aldrich). Growing colonies were gathered from the LJ medium and utilized to prepare a suspension with a concentration of 1.5 × 10^5^ colony-forming units (CFU/ml) for wells inoculation. A volume of 100 μl of 7H9 medium containing OADC was dispersed in 96-well microtiter plates. For each antibiotic, serial concentrations were established according to Table 3, and then 100 μl of bacterial suspension was added to each well. Parafilm and zip lock bags were employed to keep the microplates from drying out during the 2-week incubation period at 37°C. The MIC is defined as a drug concentration that suppresses bacterial growth by approximately 100% macroscopically. Standard strains of *Staphylococcus aureus* ATCC 2921, *Pseudomonas aeruginosa* ATCC 27853, and *Mycobacterium perginum* ATCC 700686 have been used as quality control according to CLSI recommendations [20].

**Table 3:** Susceptibility of the *M*. *simiae* isolates to 8 antimicrobial agents determined by the microbroth dilution method.

Bacterium (no. of isolates tested) and antimicrobial	Range (μg/mL)	MIC(μg/mL)		No (%) of isolates		MIC indicating Resistance (μg/mL) according to CLSI
N = 53		50%	90%	susceptible	Resistant	
Rifampin	0.5–256	64	128	0	53(100%)	>1
Isoniazid	0.5–256	64	128	0	53(100%)	>1
Clarithromycin	0.25–64	8	32	45(85%)	8(15%)	>16
Moxifloxacin	0.25–64	2	16	35(66%)	18(34%)	>2
Amikacin	0.125–64	0.5	1	53(100%)	0(0%)	>32
Clofazimine	0.25–64	32	64	0	53(100%)	>2
Linezolid	0.5–128	32	64	13(24%)	40(76%)	>16
Ethambutol	0.5–64	8	32	19(36%)	34(64%)	>4

### Analysis of mutation in drug resistance-related genes

The point mutations o]f *gyrA*, *gyrB*, and *rrl* genes were investigated in moxifloxacin and clarithromycin resistant strains of *M*. *simiae*, by PCR-sequencing method. The *gyrA* and *rrl* primers were specifically designed for this assay and the *gyrB* primer was used as described earlier [21], and are listed in Table 2. PCR amplification was carried out in a final volume of 50 μl comprising 10× PCR buffer (5 μL), MgCl2 (1.5 mM), each primer (0.2 μM), deoxynucleotide triphosphate (dNTP; 0.2 mM), Taq polymerase (2.5 unit), and 10 ng template DNA (5 μL). Amplification was performed by a thermal gradient cycler (Eppendorf Co, Hamburg, Germany). The PCR cycling conditions for each gene are as below.

*gyrA*: initial denaturation at 95°C for 10 min, followed by 30 cycles of denaturation at 95°C for 30 s, annealing at 69°C for 30 s, and a final extension at 72°C for 60 s. *gyrB*: initial denaturation at 95°C for 5 minutes and 30 cycles of amplification at 95°C for 1 minute, 64°C for 30 s, and 72°C for 1minute, followed by a final extension at 72°C for 5 minutes. *rrl*: initial denaturation at 95°C for 5 minutes and 30 cycles of amplification at 95°C for the 30s, 63°C for 30 s, and 72°C for 1minute, followed by a final extension at 72°C for 5 minutes. The expected sizes of PCR amplicons were demonstrated by electrophoresis on 1.5% horizontal agarose gel in Tris-borate-EDTA (TBE) buffer and stained with the SYBR® Safe DNA Gel Stain (Thermo Fisher Scientific) [18]. An ABI PRISM 7500 Sequence Detection System was used to determine the sequences of the PCR products (Applied Biosystems, Foster City, CA, United States). Each sequence was compared with the published gene sequence *M*. *simiae* reference strain JCM12377 in NCBI (blastN, blastX) by the MEGAX databases.

### Data analysis

The statistical data (expressed as a percentage) of resistant and sensitive bacteria to each antibiotic were performed using Statistical Package for the Social Sciences (SPSS ™) software version 22.0 (IBM Corporation, Armonk, NY, USA). The results are presented as descriptive statistics in terms of relative frequency. Whole-genome sequencing data (identification species and point mutations) were analyzed by bioinformatics tools including MEGAX software. Finally, the result was presented using tables.

## Results

**Fig 1:**
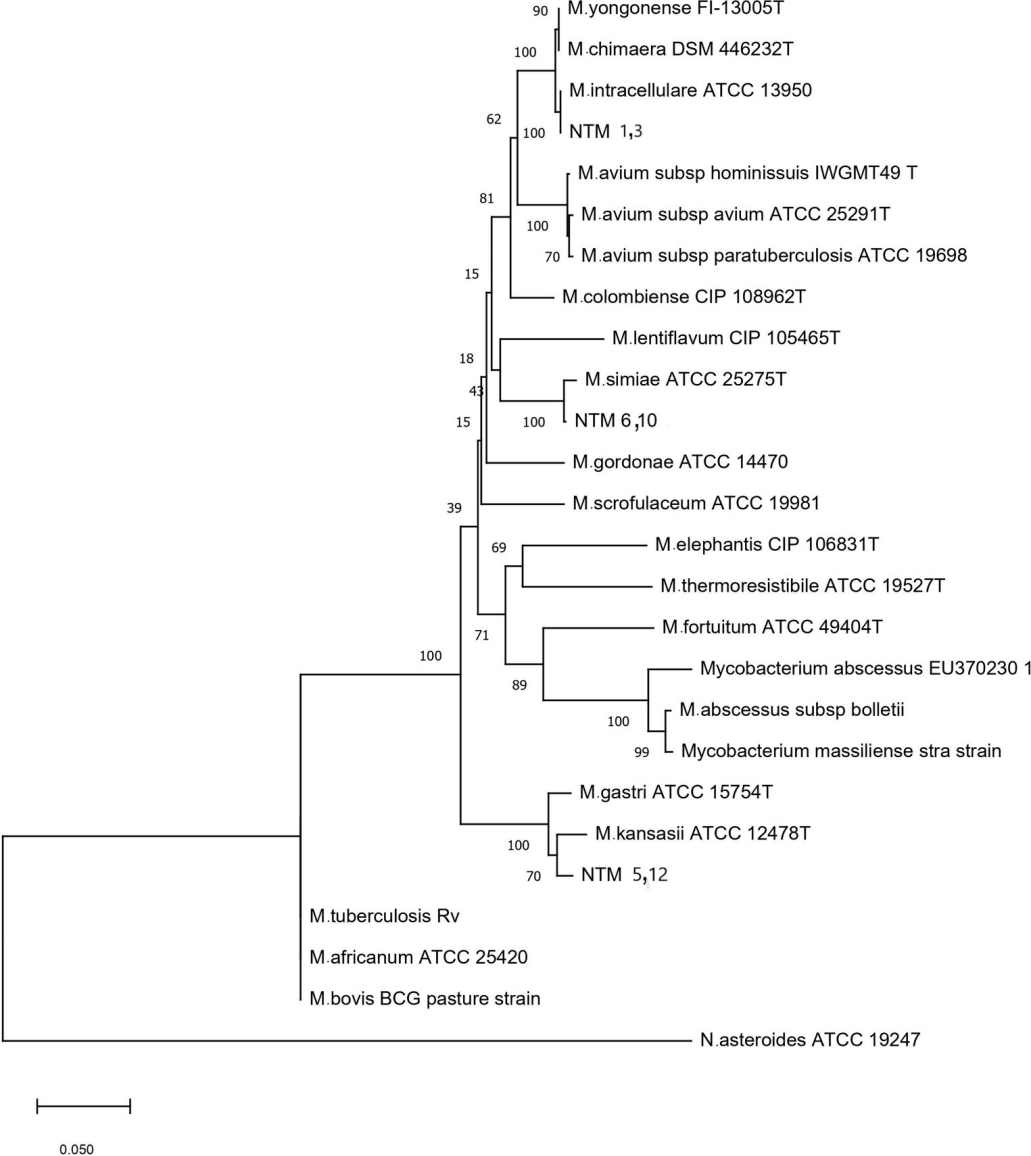
Phylogenetic tree based on *rpoB* gene sequences. *rpoB* sequence-based phylogenetic tree of the clinical isolates of NTM with those of closely related species which computed by the NJ analyses and K2P model. The support of each branch, as determined from 1000 bootstrap samples, is indicated by percentages at each node. Bar 0.01 substitutions per nucleotide position.

**Fig 2:**
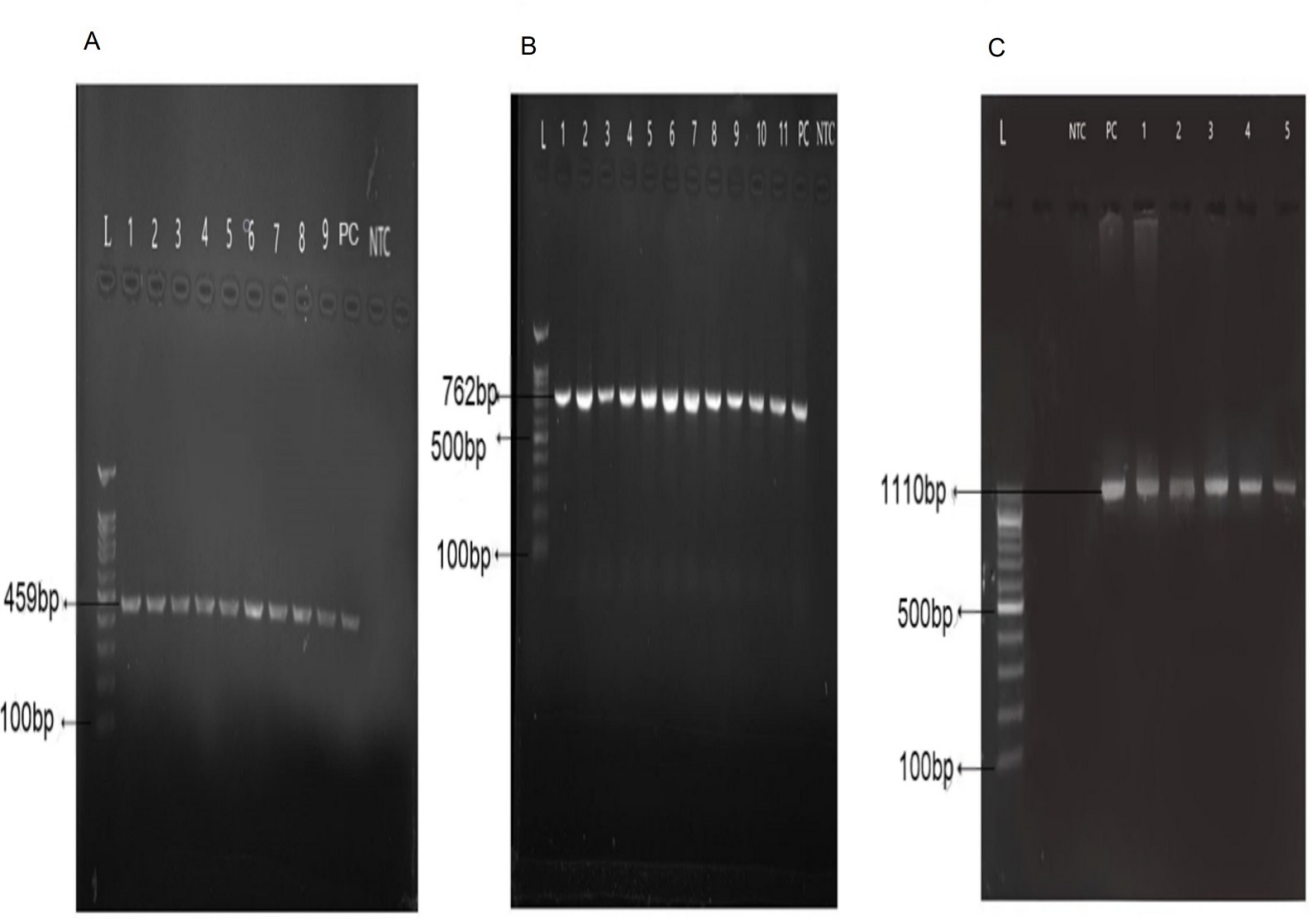
Agarose gel electrophoresis of amplified *gyrA*, *gyrB* and *rrl* genes. DNA ladder was used to proximate the gene sizes in agarose gel electrophoresis. (NTC) no template control, (PC) positive control. (A) the amplification of *gyrA* gene (459 bp), (B) the amplification of *gyrB* gene (762 bp) and (C) the amplification of *rrl* gene (1110bp).

In the current study, 50 specimens were collected from patients suffering from NTM infections. The patient’s ages ranging was from 50 to 61 years with a mean of 55.3 years. Out of 50 clinical isolates of NTM, 25 isolates were identified as *M*. *simiae* using phenotypic characteristics and biochemical tests. For definitive identification, all 25 isolates, were evaluated by *rpoB* gene sequencing, of which, 18 isolates showed more than 99% homology with *M*. *simiae* and were confirmed in the study (Table 4). The neighbor-joining phylogenetic tree based on *rpoB* sequences of isolates is illustrated in Fig 1. 35 isolates of *M*. *simiea* from the department archive belonging to previous works, were also included in the study to achieve a total of 53 *M*. *simiae* isolates for the next step of the work i.e. antibiotic susceptibility testing. The results of DST, the MIC range and MIC50, MIC90 (inhibit the growth of 50% and 90% of isolates, respectively) of 53 *M*. *simiae* isolates by microbroth dilution method are shown in Table 3. The DST of isolates was determined based on the MIC breakpoints (mg/mL) for each antibiotic published by the CLSI which are shown in the same table. All of the 53 isolates were resistant (100%) to isoniazid, rifampin, and clofazimine. The rate of resistance to ethambutol and linezolid were 34 (64%), and 40 (76%) respectively. The highest susceptibility rate was demonstrated for amikacin 53 (100%) and clarithromycin 45(85%), followed by moxifloxacin 35(66%). The MIC of each isolate against the antibiotics tested is shown in Table 5. The rate of drug resistance in *M*. *simiea* isolates against eight drugs, in tuberculosis centers of Khuzestan, Kermanshah, Fars, and Tehran provinces is shown in Table 6. Point mutations in regions of the *rrl* gene encoding the peptidyl transferase domain of the 23S rRNA cause clarithromycin resistance. Amplification of the *rrl* gene resulted successfully in an 1110 bp PCR product (Fig 2). Direct sequence analysis in eight clarithromycin-resistant isolates showed a mutation in three strains at position A2059G and one strain at position A2058G. These four strains showed high MIC resistance (MIC ≥ 64 μg/mL). In the other four resistant strains, no mutations were observed and they showed lower MIC values (MIC = 32 μg/mL). In *M*. *simiae* clinical isolates, *rrl* mutations correlated with high-level clarithromycin resistance. Genes sequences were analyzed for mutations in the quinolone-dependent region (QRDR) of the gyrase A (*gyrA*) and gyrase B (*gyrB*) genes in 18 moxifloxacin-resistant strains. Amplification of the *gyrA*, *gyrB* genes resulted successfully in a 459 bp and 762 bp PCR product (Fig 2). The results showed that no mutations were observed in the *gyrA* gene sequences. *gyrB* peptide sequence analysis indicated non-synonymous mutation of isoleucine to leucine at codon 389 (A to C, T to G) in 12 (66%) of the mutated strains. Also, in one isolate (NTM 23), a non-synonymous mutation of lysine to asparagine at codon 571(G to T), and NTM74 isolate, a mutation at codon 444 (G to C) converted the amino acid glycine to alanine (Table 7). These findings demonstrate that strains might become more resistant as a consequence of mutation.

**Table 4:** Results of *M*. *simiae* identification by phenotypic characteristics and molecular tests.

Isolate No	Pigment production	Growth rate (days)	Growth at 37°C	Colony morphology	Tween 80 hydrolysis	Arylsulfatase	Urease	Nitrate reduction	Stable heat catalase	Niacin production	Semi-quantitative catalase	Phenotypic tests	Identification by *rpoB*
**NTM1**	+	>7	+	S	-	-	+	-	+	+/-	-	*M*. *simiae*	*M*. *Intracellular*
**NTM2**	+	>7	+	S	+	-	-	-	-	-	+	*MAC*	*-*
**NTM3**	+	>7	+	S	-	-	+	-	+	+	-	*M*. *simiae*	*M*. *Intracellular*
**NTM4**	+	>7	+	S	+	-	-	-	-	-	+	*MAC*	*-*
**NTM5**	+	>7	+	S	-	-	+	-	+	+/-	-	*M*. *simiae*	*M*. *kansasii*
**NTM6**	+	>7	+	S	-	-	+	-	+	+	-	*M*. *simiae*	*M*. *simiae*
**NTM10**	+	>7	+	S	-	-	+	-	+	+	-	*M*. *simiae*	*M*. *simiae*
**NTM11**	+	>7	+	S/R	+	-	+	+	+	-	-	*M*. *kansasii*	*-*
**NTM12**	+	>7	+	s	-	-	+	-	+	+/-	-	*M*. *simiae*	*M*. *kansasii*
**NTM13**	+	>7	+	S/R	+	-	+	+	+	-	-	*M*. *kansasii*	*-*
**NTM14**	+	>7	+	S	-	-	+	-	+	+	-	*M*. *simiae*	*M*. *kansasii*
**NTM16**	+	>7	+	S	-	-	+	-	+	+	-	*M*. *simiae*	*M*. *simiae*
**NTM17**	+	>7	+	S	-	-	+	-	+	+	-	*M*. *simiae*	*M*. *simiae*
**NTM20**	+	>7	+	S	-	-	+	-	+	+	-	*M*. *simiae*	*M*. *simiae*
**NTM23**	+	>7	+	S	-	-	+	-	+	+	-	*M*. *simiae*	*M*. *simiae*
**NTM24**	+	>7	+	S	-	-	+	-	+	+	-	*M*. *simiae*	*M*. *kansasii*
**NTM25**	+	>7	+	S	-	-	+	-	+	+	-	*M*. *simiae*	*M*. *Intracellular*
**NTM26**	+	>7	+	S	+	-	-	-	-	-	+	*MAC*	*-*
**NTM27**	+	>7	+	S	-	-	+	-	+	+	-	*M*. *simiae*	*M*. *simiae*
**NTM28**	+	>7	+	S	-	-	+	-	+	+	-	*M*. *simiae*	*M*. *simiae*
**NTM30**	+	>7	+	S/R	+	-	+	+	+	-	-	*M*. *kansasii*	*-*
**NTM32**	+	>7	+	S/R	+	-	+	+	+	-	-	*M*. *kansasii*	*-*
**NTM34**	+	>7	+	S	+	-	-	-	-	-	+	*MAC*	*-*
**NTM35**	+	>7	+	S	+	-	-	-	+	-	+	*MAC*	*-*
**NTM36**	+	>7	+	S	-	-	+	-	+	+	-	*M*. *simiae*	*M*. *simiae*
**NTM38**	-	<7	+	S/R	+	+	+	+	+	-	-	*M*. *fortuitum*	*-*
**NTM40**	+	>7	+	S	+	-	-	-	-	-	+	*MAC*	*-*
**NTM41**	+	>7	+	S	+	-	-	-	-	-	+	*MAC*	*-*
**NTM43**	+	>7	+	S	-	-	+	-	+	+	-	*M*. *simiae*	*M*. *simiae*
**NTM49**	+	>7	+	S	-	-	+	-	+	+	-	*M*. *simiae*	*M*. *simiae*
**NTM53**	+	>7	+	S	-	-	+	-	+	+	-	*M*. *simiae*	*M*. *simiae*
**NTM54**	+	>7	+	S	-	-	+	-	+	+	-	*M*. *simiae*	*M*. *simiae*
**NTM57**	+	<7	+	S	+	-	+	-	+	-	-	*M*. *fortuitum*	*-*
**NTM58**	-	<7	+	S	+	+	+	+	+	-	-	*M*. *fortuitum*	*-*
**NTM59**	-	<7	+	S	+	+	+	+	+	-	-	*M*. *fortuitum*	*-*
**NTM63**	+	>7	+	R	-	-	+	-	+	-	-	*M*. *scrofulaceum*	*-*
**NTM64**	-	<7	+	S/R	+	+	+	+	+	-	-	*M*. *fortuitum*	*-*
**NTM85**	+	>7	+	S	-	-	+	-	+	-	-	*M*. *scrofulaceum*	*-*
**NTM86**	-	<7	+	S/R	+	+	+	+	+	-	-	*M*. *fortuitum*	*-*
**NTM87**	+	>7	+	S	-	-	+	-	+	+	-	*M*. *simiae*	*M*. *simiae*
**NTM96**	+	>7	+	S	-	-	+	-	+	+	-	*M*. *simiae*	*M*. *simiae*
**NTM99**	+	>7	+	S	-	-	+	-	+	+	-	*M*. *simiae*	*M*. *simiae*
**NTM100**	+	>7	+	S	-	-	+	-	+	+	-	*M*. *simiae*	*M*. *simiae*
**NTM102**	+	>7	+	S	-	-	+	-	+	+	-	*M*. *simiae*	*M*. *simiae*
**NTM103**	+	>7	+	S	+	-	-	-	-	-	+	*MAC*	*-*
**NTM104**	+	>7	+	S	+	-	-	-	-	-	+	*MAC*	*-*
**NTM110**	+	>7	+	S/R	+	-	+	+	+	-	-	*M*. *kansasii*	*-*
**NTM116**	+	>7	+	S/R	+	-	+	+	+	-	-	*M*. *kansasii*	-
**NTM136**	+	>7	+	S/R	+	-	+	+	+	-	-	*M*. *kansasii*	-
**NTM137**	+	>7	+	S/R	+	-	+	+	+	-	-	*M*. *kansasii*	*-*

S: Smooth / R: Rough

**Table 5:** Minimum inhibitory concentration of *M*. *simiae* isolates.

	Isolate	AMK (μg/mL)	MOX (μg/mL)	CLR (μg/mL)	CLO (μg/mL)	LIN (μg/mL)	RIF (μg/mL)	INH (μg/mL)	EB (μg/mL)
**1**	NTM6	0.5	2	4	16	8	64	64	8
**2**	NTM10	0.5	8	2	8	64	64	256	8
**3**	NTM16	0.5	2	16	16	32	64	128	32
**4**	NTM17	0.5	2	4	16	16	64	64	16
**5**	NTM20	1	16	16	16	64	64	64	16
**6**	NTM23	0.5	16	32	16	64	128	32	32
**7**	NTM27	0.5	1	16	8	8	128	64	8
**8**	NTM28	1	8	8	16	32	128	128	8
**9**	NTM33	1	1	4	8	64	128	64	16
**10**	NTM36	0.5	1	4	32	64	256	128	4
**11**	NTM37	0.5	0.5	8	16	32	256	128	16
**12**	NTM43	0.5	2	2	8	32	64	32	4
**13**	NTM44	0.5	16	16	8	32	128	64	8
**14**	NTM49	0.5	1	4	16	32	256	32	2
**15**	NTM50	0.5	16	8	32	64	64	64	4
**16**	NTM53	1	2	1	32	8	128	64	4
**17**	NTM54	0.5	2	2	16	32	128	64	32
**18**	NTM68	0.5	16	1	8	64	256	128	4
**19**	NTM74	1	16	64	8	16	128	128	4
**20**	NTM78	0.5	0.5	16	8	32	128	128	16
**21**	NTM83	0.5	2	8	32	64	128	64	8
**22**	NTM87	0.5	0.5	8	32	8	64	32	32
**23**	NTM96	1	0.5	64	64	32	64	64	8
**24**	NTM99	1	0.5	4	64	64	256	128	8
**25**	NTM100	0.5	32	64	32	64	128	64	4
**26**	NTM102	0.5	32	32	32	32	128	128	4
**27**	NTM105	0.5	2	4	32	32	64	64	16
**28**	NTM106	0.25	2	64	64	32	128	32	4
**29**	NTM109	0.5	1	16	64	32	64	64	8
**30**	NTM300	0.5	2	8	32	64	128	128	4
**31**	NTM302	0.5	8	2	64	8	64	32	16
**32**	NTM307	0.25	1	4	16	64	128	64	32
**33**	NTM314	0.5	0.5	2	32	64	64	64	16
**34**	NTM315	0.25	8	16	32	64	32	64	2
**35**	NTM319	1	1	8	16	64	128	32	4
**36**	NTM335	0.5	16	4	32	16	32	32	8
**37**	NTM339	0.5	2	32	16	8	64	32	16
**38**	NTM341	1	1	8	64	32	32	64	32
**39**	NTM342	1	16	8	32	64	64	128	64
**40**	NTM343	1	16	8	64	64	32	128	8
**41**	NTM347	0.25	2	8	64	32	64	64	2
**42**	NTM350	0.5	2	8	32	32	32	256	8
**43**	NTM370	0.5	2	32	32	32	64	64	4
**44**	NTM373	0.5	0.5	8	32	32	32	32	8
**45**	NTM378	0.5	1	2	16	64	128	64	4
**46**	NTM381	0.25	2	8	8	8	64	128	1
**47**	NTM389	0.5	32	8	8	16	64	128	2
**48**	NTM394	0.5	2	8	32	64	128	128	32
**49**	NTM397	0.5	1	4	16	32	128	256	64
**50**	NTM403	1	1	16	64	16	128	64	16
**51**	NTM415	0.5	4	8	16	64	32	128	8
**52**	NTM416	0.25	16	2	64	8	64	256	4
**53**	NTM421	1	2	4	32	32	32	64	8

AMK = Amikacin, MOX = Moxifloxacin, CLR = Claritromycin, CLO = Clofazimine, LIN = linezolid, RIF = Rifampin, INH = Isoniaside, EB = Ethambutol

**Table 6:** The regions and geographic distribution of drug-resistant *M*. *simiae* strains.

	Tehran	Khuzestan	Fars	Kermanshah
**Isolates**	10(18.86%)	18(34%)	12(22.6%)	13(24.54%)
**Rifampin**	10(100%)	18(100%)	12(100%)	13(100%)
**Isoniazid**	10(100%)	18(100%)	12(100%)	13(100%)
**Ethambutol**	7(70%)	9(50%)	8(66.7%)	10(77%)
**Clofazimine**	10(100%)	18(100%)	12(100%)	13(100%)
**Clarithromycin**	3(30%)	2(11%)	3(25%)	0(0%)
**Amikacin**	0(0%)	0(0%)	0(0%)	0(0%)
**Moxifloxacin**	3(30%)	6(33%)	4(33.3%)	5(38.4%)
**Linezolid**	8(80%)	15(83%)	7(58.3%)	10(77%)

**Table 7:** Mutation patterns against clarithromycin and moxifloxacin resistance strains.

drugs	Acquired mutations in target gene	position	mutant	codon	Amino acid
**clarithromycin**	*rrl*	2058	A G	-	-
		2059	A C	-	-
**moxifloxacin**	*gyrB*	1165	A C	389	Isoleucine Leucine
		1167	T G		
		1713	G T	571	Lysine Asparagine
		1304	G C	444	Glycine Alanine

Adenine (A), Cytosine (C), Guanine (G), Thymine (T)

## Discussion

Identification of NTM to the species level is an important issue in determining the appropriate antibiotic regimen for the treatment and requires the use of efficient and accurate methods [22, 23]. Advances in molecular methods have helped the precise identification of NTM species in recent years. Sequence-based methods are one of the definitive methods for identifying NTM to distinct species [24, 25]. As reported by Heidarieh et al. [26], *M*. *simiae* is one of the three common isolated species among NTM in Iran. Antibiotic resistance in *M*. *simiae* is increasing recently [26]. Although treatment of NTM diseases has generally been established based on expert opinions and using drugs available in standard doses [23], however, still no standard treatment regimen is available for *M*. *simiae* infection. Mechanisms of resistance, such as mutations detection in regions that affect resistance, are an important issue that has not yet been extensively studied [10, 23].

In the present study, we applied phenotypic and molecular methods to identify *M*. *simiae* isolates. Based on the *rpoB* gene sequencing and PCR method, 18 isolates were identified as *M*. *simiae* species, whereas 25 isolates were identified using phenotypic methods, indicating that sequence-based in recognizing NTM provides significantly greater resolution than the phenotypic approaches, as demonstrated in several studies which similarly applied the *rpoB* gene sequencing to identify NTM species [22, 24, 27–29]. Investigation of the DST of *M*. *simiae* isolates against eight antibiotics was one of our main goals in the current study. Results of DST demonstrated that the highest resistances related to rifampin, isoniazid, and clofazimine (100%), followed by ethambutol (64%), linezolid (76%). The lowest resistance was seen for amikacin (0%), clarithromycin (15%), and moxifloxacin (34%). Earlier studies of the DST of *M*. *simiae* isolates showed, resistance (100%) to the first-line TB drugs, whereas successful treatment regimens were including macrolides, quinolones, clofazimine, and aminoglycosides [30]. A recent study showed all clinical *M*. *simiae* isolates were resistant (100%) to streptomycin, amikacin, kanamycin, ciprofloxacin, and clarithromycin in addition to the first-line TB drugs [31]. Clinical *M*. *simiae* isolates are resistant to many first-line TB drugs. Several studies have verified the high rate of resistance in *M*. *simiae* to isoniazid, rifampin, and ethambutol which was in line with our findings [32–34]. In addition, according to a report by van Ingen et al., *M*. *simiae* is resistant to rifampicin and ethambutol alone and in combination [35]. A study by Farnia et al. suggested that first-line TB drugs should be omitted from the treatment regimen of *M*. *simiae* [36]. In our study, we reported resistance to clarithromycin and moxifloxacin at 15% and 34% respectively. In the same line, Lotfi et al. [5] In 2021 report of the DST results for a single isolate of *M*. *simiae*, showed resistance to moxifloxacin and clarithromycin as well [5]. Additionally, they showed *M*. *simiae* were sensitive to amikacin, which was concordant with our findings, however against our results, they reported sensitivity to clofazimine. More in agreement with our findings, In the study by Karami-Zarandi et al. from Tehran [32], their 17 *M*. *simiae* strains showed high resistance to linezolid (94%), rifampin (94%), and isoniazid (100%). In our study, all *M*. *simiae* isolates were resistant (100%) to three drugs, while the most effective antimicrobial agents against *M*. *simiae* isolates were amikacin and clarithromycin, which in the aforesaid study [32], resistance to clarithromycin and amikacin were 58%, 47%, respectively, which was higher than our results. In the study conducted in Lebanon by Hamieh et al. [13], similar to our outcomes, amikacin and clarithromycin were identified as the most effective antibiotics with the susceptibility rate of 88.3% and 94.2% respectively. There are other similar reports regarding the effectiveness of moxifloxacin and clarithromycin against *M*. *simiae*. van Ingen et al. from UK [35], evaluated the sensitivity of *M*. *simiae* complex to thirteen drugs. All of 22 *M*. *simiae* strains showed the highest level of drug resistance *in vitro*. The most effective drugs were clarithromycin (9%) and moxifloxacin (36%), furthermore, resistance to clofazimine, amikacin, and linezolid were 55%, 86%, and 100%, respectively in their study. In this regard, Coolen-Allou et al. [25], in France reported susceptibility to amikacin, moxifloxacin, ciprofloxacin, and clarithromycin were 96%, 92%, 87%, and 100%, respectively. The isolates were more susceptible to moxifloxacin and clarithromycin, compared to our study. Differences in drug susceptibility to some antibiotics may be due to the variable origin and various isolated sources of *M*. *simiae*.

Nowadays, the molecular mechanisms that cause natural and acquired resistance to antibiotics have been considered. It is possible to identify resistance by sequencing known genes involved in resistance [37]. According to our findings, *gyrB* peptide-sequencing showed mutations in codons 389, 444, and 571. No mutations were observed in the *gyrA* gene-sequencing analysis and sequencing of the *rrl* gene showed point mutations in positions 2058 and 2059. However, in contrast to our results, Lotfi et al. [5], showed deletion in the bases 1148, 1149, and 1150, and the amino acid phenylalanine was removed and replaced with a stop codon also at position 1066, and the amino acid glutamate was converted to serine in the *gyrA* gene. In their study, mutations were identified at position 442 in the *gyrB* gene and location 217 in the *rrl* gene. There are a few studies on molecular detection of mutations in *M*. *simiae*. Our study is one of the first studies to investigate genomic mutations related to drug resistance in *M*. *simiae*. Identifying and exploring the link between genomic mutations and drug resistance in *M*. *simiae* can help to control infection. Extensive research is required to investigate the causes of drug resistance in this bacterium. In conclusion evaluation of drug susceptibility *in vitro* showed that *M*. *simiae* is highly resistant to antibiotics. Amikacin, clarithromycin, and moxifloxacin were the most effective drugs against *M*. *simiae*. Also, genomic mutations in resistant strains played an important role in causing high MIC. This investigation has some limitations such as time constraints by the Covid-19 pandemic to sample collection and a lack of financial resources to assess further drug resistance genes. future research can focus on finding effective antibiotics and evaluating the other resistance genes to greatly help in preventing the spread of antibiotic resistance.
